# Mesenchymal stromal cell treatment improves outcomes in children with pneumonia post-hematopoietic stem cell transplantation: a retrospective cohort study

**DOI:** 10.1186/s13287-022-02960-7

**Published:** 2022-06-28

**Authors:** Yuhua Qu, Xu Yang, Xiaohong Zhang, Shanshan Liu, Xiaoping Liu, Xiaodan Liu, Ailing Luo, Mansi Cai, Yaping Yan, Ling Xu, Hua Jiang

**Affiliations:** Department of Hematology, Guangzhou Women and Children’s Medical Center, Guangzhou Medical University, Guangzhou, 510623 Guangdong China

**Keywords:** Mesenchymal stromal cell (MSC), Hematopoietic stem cell transplantation (HSCT), Pneumonia, Severe pneumonia

## Abstract

**Background:**

Hematopoietic stem cell transplantation (HSCT) is a standard therapy strategy for most malignant disorders in children. However, transplant-related pneumonia remains a major therapy challenge and mesenchymal stromal cells (MSCs) are rarely reported in HSCT-related pneumonia. The aim of our study was to assess the efficacy of MSC for HSCT-related pneumonia in children.

**Methods:**

We retrospectively retrieved HSCT-related (severe and non-severe) pneumonia cases (aged < 18 years), which underwent MSC treatment (MSC group) or non-MSC treatment (non-MSC group) in Guangzhou Women and Children’s Medical Center, from December 2017 to December 2019. We investigated outcomes of the two different treatments among severe cases and non-severe cases, respectively. The primary endpoints were differences in overall cure rate and time to cure between MSC and non-MSC groups. The secondary endpoints were 180-day overall survival and cumulative cure rate.

**Results:**

Finally, 31 severe pneumonia cases (16 in MSC group, 15 in non-MSC group) and 76 non-severe cases (31 in MSC group, 45 in non-MSC group) were enrolled in this study. Among severe pneumonia cases, overall cure rate in MSC group was significant higher than that in non-MSC group (12[75.0%] vs. 5[33.3%]; OR = 6.00, 95% CI [1.26–28.5]; *p* = 0.020); the time (days) to cure in MSC group was dramatically reduced compared with that in non-MSC group (36 [19–52] vs. 62 [42–81]; OR = 0.32, 95% CI [0.12–0.88]; *p* = 0.009); the 180-day overall survival in MSC group was better than that in non-MSC group (74.5% [45.4–89.6] vs. 33.3% [12.2–56.4]; *p* = 0.013). Among non-severe pneumonia cases, the time (days) to cure in MSC group was notably decreased compared with that in non-MSC group (28 [24–31] vs. 33 [26–39]; OR = 0.31, 95% CI [0.18–0.56]; *p* = 0.003). Compared with non-MSC group, MSC-treated patients achieved significant improvements of cumulative cure rate not only in severe pneumonia cases (*p* = 0.027), but also in non-severe cases (*p* < 0.001).

**Conclusions:**

This study revealed that MSC treatment could contribute to improving outcomes in children with pneumonia post-HSCT, especially in severe cases. These findings suggest MSC treatment as a promising therapy for HSCT-related pneumonia in children.

**Supplementary Information:**

The online version contains supplementary material available at 10.1186/s13287-022-02960-7.

## Introduction

Hematopoietic stem cell transplantation (HSCT) is a standard therapy strategy for most hematologic disorders and malignancies in children. Around the world, there are more than fifty thousand HSCTs performed each year [1, 2]. Despite the improved survival attributed to advances in HSCT including transplantation techniques and supportive care, transplant-related pneumonia (severe pneumonia in particular) remains the leading cause of death for most HSCT patients and a major challenge for clinicians.

Transplant-related pneumonia, including severe and non-severe conditions, occur in more than one third of patients undergoing HSCT and account for a significant proportion of mortality in HSCT recipients [3, 4]. Considering the lung tissue damage and immune response state of HSCT patients with pneumonia, immunoregulation, and tissue repair are the main challenges in the therapy for pneumonia. Given the limitations of conservative treatment, cell therapy is being developed as a new strategy. Mesenchymal stromal cells (MSCs), a form of multipotent cells, have been applied in therapy for various intractable disorders. MSC exerts its therapeutic effect through various biological functions including immunoregulation, tissue repairing, self-renew, and differentiating into various cell lines [5, 6]. For example, MSC has been demonstrated to contribute to lung tissue regeneration and remodeling, decrease inflammation, and restore lung fluid balance [6–8]. The American Thoracic Society has also suggested MSCs as a cell therapy agent for lung diseases [9, 10]. However, MSC therapy for pneumonia following HSCT has not been well investigated. MSCs can be isolated from many tissues, including the umbilical cord (UC), bone marrow (BM), and adipose tissue (AT) [5, 11].

In this retrospective study, we assessed the efficacy of a UC-MSC-based therapy for HSCT-related pneumonia in children and found the MSC-based treatment significantly increased the cure rate and decreased the mortality in HSCT patients with severe pneumonia. Our results demonstrate the significant therapeutic benefit of MSC treatment for HSCT-related pneumonia and might provide a promising alternative therapy for severe pneumonia.

## Methods

### Study design and subjects

In this retrospective study, we reviewed patients who underwent HCST between December 2017 and December 2019, in Guangzhou Women and Children’s Medical Center (GWCMC), and retrieved those cases that got pneumonia after HSCT. The study was registered at ClinicalTrials.Gov (NCT05131412). The key inclusion criteria were: (1) patients with pneumonia after HSCT; (2) patients under the age of 18 years; and (3) patients with normal pulmonary function before HSCT. The excluded cases were patients with other severe complications in progress when pneumonia occurred. Clinical characteristics and laboratory findings were also retrieved from the electronic medical record (EMR) system of GWCMC. All patients were followed up for at least 12 months, unless the patient died or was lost to follow-up. We obtained written informed consent for all patients from their parents or legal guardians before they were enrolled in the study. All procedures in this study were performed in accordance with the guidelines of Institutional Review Board of GWCMC.

### Procedures

According to information of the EMR system, the included cases were classified into two treatment groups (MSC group and non-MSC group) based on whether MSC was used in the therapy for HSCT-related pneumonia. The two groups were enrolled contemporaneously, and group allocation depended on the decision of the parents or guardians. The MSCs used in this study were umbilical cord (UC)-MSC products (Qilu Cell Therapy Engineering & Technology Co., Ltd., Jinan, Shandong, China). Every UC-MSC product was from an individual donor and sorted by MSC markers (CD90^+^, CD44^+^, CD73^+^, CD105^+^, CD34^−^, and CD45^−^) with flow cytometry. Before used, every UC-MSC product met all of the following conditions: (1) negative in the detection of microorganism (including bacteria, fungi, and mycoplasma); (2) with the concentration of endotoxin ≤ 0.5 endotoxin unit (EU)/mL; and (3) cell viability ≥ 90%. In the non-MSC group, patients were treated with conventional strategies: (1) antibiotics, (2) immunosuppressive agent, (3) anti-virus or antifungal agents, if serology was positive for viral or fungal agents. In the MSC group, MSC was administered in combination with a modified conventional strategy, in which the immunosuppressive agent was taken at a half dose, and antibiotics, antivirals, and antifungals were given the same way as in the non-MSC group. In previous studies [12–15], MSCs were used at 1–2 × 10^6^ cells/kg body weight in most cases, and it was safe and feasible to use in weekly. In this study, MSCs were infused intravenously once a week at a dose of 1 × 10^6^ cells/kg body weight, until a cure was achieved or mortality occurred. Pneumonia was categorized into severe and non-severe (mild) subtypes according to the medical records of GWCMC based on the guidelines for childhood pneumonia [16].

### Outcomes

The primary endpoints were overall cure rate and time to cure. The cure was defined as the complete disappearing of pneumonia diagnosed by pediatricians based on the clinical and laboratory findings. Time to cure was defined as the time from the first diagnosis of pneumonia to a cure was achieved. The secondary endpoints were overall survival and cumulative cure rate. Overall survival was defined as the time from pneumonia occurred to death from any cause. Surviving patients or those lost to follow-up were censored at last follow-up.

### Statistical analysis

In the analysis of patient characteristics, continuous variables between different groups were evaluated with two-independent sample t test in the form of mean ± SD. Qualitative variables were described as numbers (%) and were assessed using the goodness-of-fit Chi-square (*χ*^2^) test or the Fisher’s exact test. Differences between MSC and non-MSC groups in overall cure rate were analyzed by Fisher’s exact test. Odds ratio (OR) with corresponding 95% confidence interval (CI) was assessed using Woolf and Baptista–Pike method as appropriate. The comparisons of time to cure between groups were estimated with Mann–Whitney test. The different treatment groups were examined as MSC versus non-MSC in all comparisons. Overall survival and cumulative cure rates were estimated using Kaplan–Meier method, and differences between groups were compared with the log-rank test. The above analyses were performed among severe cases, non-severe cases, and total cases, respectively. In the present study, all *P* values were two-sided and values of *P* < 0.05 were considered statistically significant. Statistical analyses were performed with the SPSS Statistics 24 software, unless otherwise indicated.

## Results

### Subjects

As shown in Table 1, a total of 107 cases, including 47 cases in MSC group and 60 cases in non-MSC group, were enrolled in the study, based on the above inclusion and exclusion criteria. Most of them were thalassemia cases, other cases included mucopolysaccharidoses (MPS), adrenoleukodystrophy (ALD), pyruvate kinase deficiency (PKD), hyperimmunoglobulin E syndrome (HIES), and leukemia. The mean ages of the MSC group and the non-MSC group were 6.11 (± 2.91) years and 6.41 (± 2.96) years, respectively. MSC group consist of 31 male and 16 female children, and non-MSC group consist of 38 male and 22 female children. There were 16 severe pneumonia cases and 31 non-severe cases in MSC group, and there were 15 severe pneumonia cases and 45 non-severe cases in non-MSC group. Donor type, stem cell source, pathogen infection status, and other clinical characteristics are also displayed in Table 1. All the above characteristics between the two groups are similar (all *p* > 0.05). In the MSC-treatment group, mean MSC infusion times were 4.02 ± 2.11 (median: 4, range 1–11): 5.25 ± 2.44 (median: 5, range 2–11) for severe pneumonia cases and 3.42 ± 1.63 (median: 3, range 1–9) for non-severe cases. Notably, among all of the pneumonia patients, only 39 (36.44%) cases were virus positive in serostatus (18 cases in MSC group, 21 cases in non-MSC group). In addition, 12 (11.21%) cases were virus positive in bronchoalveolar lavage fluid (BALF) but negative in serostatus. In total, the overall proportion of virus-positive cases was only 47.65%. Similarly, in another study investigating pathogen by metagenomics sequencing, we detected 37 (50.00%) virus-positive cases in 74 HSCT-related pneumonia patients (unpublished data). These results implied that a large portion of patients were virus negative or some virus-infected patients could not be diagnosed. Moreover, compared with antiviral treatment, immune disorder is considered as the key symptom for all patients with or without virus infection, and more attention should be paid to immunoregulation.

**Table 1:** Patient characteristics of MSC group and non-MSC group

	MSC group (*n* = 47)	Non-MSC group (*n* = 60)	*p* value
	No. (%)	No. (%)	
Age (year)			
Mean ± SD (range)	6.19 ± 3.04 (1.10–15)	6.54 ± 2.94 (1.10–15)	0.608
≤ 7	33 (70.21)	42 (70.00)	0.981
> 7	14 (29.79)	18 (30.00)	
Sex			0.778
Male	31 (65.96)	38 (63.33)	
Female	16 (34.04)	22 (36.67)	
Pneumonia			0.306
Non-severe	31 (65.96)	45 (75)	
Severe	16 (34.04)	15 (25)	
HSC source^a^			0.454
Peripheral blood	41 (87.23)	55 (91.67)	
Umbilical cord blood	6 (12.77)	5 (8.33)	
HSC donor type^b^			0.800
Matched	27 (57.45)	33(55.00)	
Mismatched	20 (42.55)	27 (45.00)	
Primary diseases			0.176
Thalassemia	36 (76.60)	52 (86.67)	
Others	11 (23.40)	8(13.33)	
Interval days^c^			
≥ 100	20 (42.55)	19 (31.67)	0.246
< 100	27 (57.45)	41 (68.33)	
Virus serostatus			0.725
Negative	29 (61.70)	39 (65.00)	
Positive	18 (38.30)	21 (35.00)	
Virus (serostatus + BAL)^d^			
Negative	23 (48.94)	33 (55.00)	0.533
Positive	24 (51.06)	27 (45.00)	
Bacteria/fungi			0.454
Negative	41 (87.23)	55 (91.67)	
Positive	6 (12.77)	5 (8.33)	
MSC infusion times			
Mean ± SD (Median, range)	4.04 ± 2.11 (4, 1–11)		

^a,b^HSC: hematopoietic stem cell

^c^Days between pneumonia onset and hematopoietic stem cell transplantation

^d^BAL: bronchoalveolar lavage fluid

### Evaluation of therapeutic effect

Abnormalities in chest CT images were detected among pneumonia cases in both MSC group (Additional file 1: Fig. S1A) and non-MSC group (Additional file 1: Fig. S1B). During the treatment for pneumonia, the fading and disappearing of abnormalities in CT images indicated a good prognosis (Additional file 1: Fig. S1A), while deteriorating CT results were detected in patients who ultimately died of pneumonia (Additional file 1: Fig. S1B).

In this current study, all patients were either cured or dead within 180 days, although the follow-up time was more than 12 months. We investigated the overall cure rates between MSC and non-MSC groups, among severe pneumonia cases, non-severe pneumonia cases, and total cases. The results (Table 2) showed that the overall cure rate (75.0%) in MSC group was higher than that (33.3%) in non-MSC groups among severe cases, and the difference was significant (OR = 6.00, 95% CI [1.26–28.5]; *p* = 0.020). However, no difference was found between groups among non-severe cases or total cases, which might be attributed to the same cure rates (100%) in both treatment groups of non-severe cases. As the other primary endpoint, time to cure was also estimated between the different treatment groups. As shown in Table 3, the differences in time (days) to cure between MSC and non-MSC groups are all remarkable among severe pneumonia cases (36 [19–52] vs. 62 [42–81]; OR = 0.32, 95% CI [0.12–0.88]; *p* = 0.009), non-severe cases (28 [24–31] vs. 33 [26–39]; OR = 0.31, 95% CI [0.18–0.56]; *p* = 0.003), and total cases (29 [25–34] vs. 44 [38–52]; OR = 0.46, 95% CI [0.29–0.73]; *p* = 0.003).

**Table 2:** Overall cure rate of MSC group versus non-MSC group

	Cure case (%)	Odd ratio (95% CI)	*p* value
Severe pneumonia			
MSC (*n* = 16)	12 (75.0)	6.00 (1.26–28.5)	0.020
Non-MSC (*n* = 15)	5 (33.3)	Ref	
Non-severe pneumonia			
MSC (*n* = 31)	31 (100.0)	–	–
Non-MSC (*n* = 45)	45 (100.0)	Ref	
All cases			
MSC (*n* = 47)	43 (91.5)	2.15 (0.629–7.345)	0.214
Non-MSC (*n* = 60)	50 (83.3)	Ref

**Table 3:** Time to cure of MSC group versus non-MSC group

	Median days (95% CI)	Odd ratio (95% CI)	*p* value
Severe pneumonia			
MSC group (*n* = 16)	36 (19–52)	0.32 (0.12–0.88)	0.009
Non-MSC group (*n* = 15)	62 (42–81)	Ref	
Non-severe pneumonia			
MSC group (*n* = 31)	28 (24–31)	0.31 (0.18–0.56)	0.003
Non-MSC group (*n* = 45)	33 (26–39)	Ref	
All cases			
MSC group (*n* = 47)	29 (25–34)	0.46 (0.29–0.73)	0.003
Non-MSC group (*n* = 60)	44 (38–52)	Ref	

**Fig. 1:**
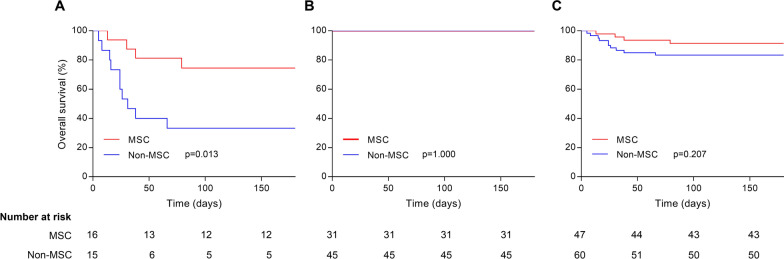
Overall survival by treatment with MSC or without MSC among severe pneumonia cases (**a**), non-severe cases (**b**), and total cases (**c**)

**Fig. 2:**
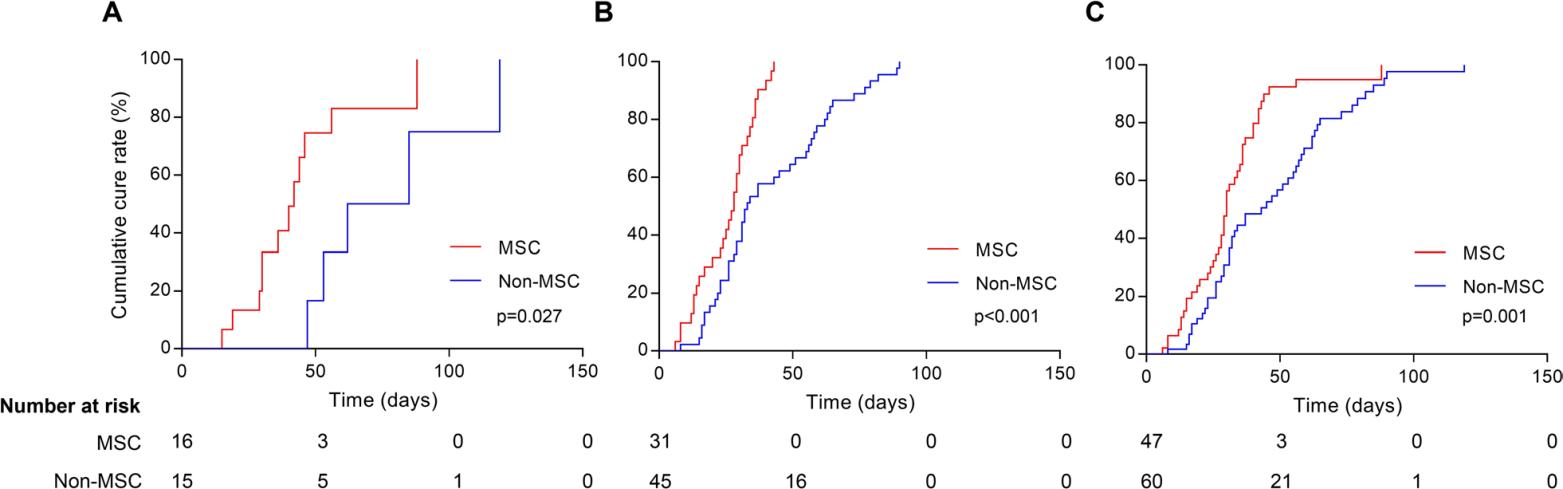
Cumulative cure rate by treatment with MSC or without MSC among severe pneumonia cases (**a**), non-severe cases (**b**), and total cases (**c**)

Furthermore, we performed analyses of overall survival and cumulative cure rate depending on time for severe pneumonia cases and non-severe cases, respectively. As shown in Fig. 1a, MSC treatment resulted in increased 180-day overall survival only for severe pneumonia cases (74.5% [45.4–89.6] vs. 33.3% [12.2–56.4]; *p* = 0.013), which means that MSC reduced severe-pneumonia-related mortality. The high mortality in control group was consistent with the reports in previous studies [17, 18]. As for non-severe cases (Fig. 1b, *p* = 1.000) and total cases (Fig. 1c, *p* = 0.207), no differences were found in mortality between MSC group and the non-MSC group, which was attributed to the survivals of 100% in both treatment groups among non-severe cases. Meanwhile, MSC-treated patients achieved significant improvements of cumulative cure rate among severe pneumonia cases (Fig. 2a, *p* = 0.027), non-severe cases (Fig. 2b, *p* < 0.001), and total cases (Fig. 2c, *p* = 0.001). In addition, one or more complications that are not secondary to severe pneumonia might occur in HSCT patients. To clarify whether MSC treatment influences the incidence of secondary disorders in posthumous cases, we estimated secondary complications during the treatment for pneumonia. We found no significant difference in incidence of complications between the MSC group and the non-MSC group, and we found that lung dysfunction secondary to severe pneumonia, including respiratory failure, was the leading cause of death in both groups (Table 4).

**Table 4:** Complications in death cases between MSC group and non-MSC group

	MSC	Non-MSC	*p* value
	*n* (%)	*n* (%)	
Severe pneumonia	4 (100)	10 (100)	0.758
Respiratory failure	4 (100)	10 (100)	
GVHD	0 (0)	2 (20)	
Organ failure	1 (25)	2 (20)	
Septicemia	1 (25)	1 (10)	
Secondary diabetes	1 (25)	0 (0)	
Shock	0 (0)	3 (30)	
Mediastinal emphysema	0 (0)	2(20)	
Non-pulmonary inflammation	1 (25)	2 ( 20)	

## Discussion

As an established, intensive therapy, HSCT provides a promising treatment for patients with various malignant disorders, yet transplant-related complications remain a challenge for physicians and patients alike. Despite the advancement in transplant techniques and supportive care, pulmonary complications, including pneumonia, present in a large proportion of HSCT recipients and cause nearly 50% of transplant-related deaths. It is urgently necessary to develop an effective therapy to improve the prognosis of HSCT-related pneumonia. MSCs, as a pluripotent cell agent, could yield a promising therapy strategy. In this study, we investigated and evaluated the efficacy of an MSC-based treatment in the therapy for pneumonia after HSCT.

Since first being tested as a cell therapy agent in humans in 1995 [19], MSCs have been widely studied and increasingly developed in preclinical and clinical applications. Initially, most MSC products were mainly bone marrow-derived, but now MSCs can be isolated from various tissues, such as the umbilical cord, adipose tissue, muscle, and bone [5, 11]. Although bone marrow-derived MSCs are the earliest discovered and most widely used, they have several limitations including invasive procedures and a significant decrease in cell number and differentiation potential with age. MSCs derived from other tissues have been discovered and isolated. UC-MSC has been demonstrated to be an excellent alternate [20–22]. Compared with BM-MSC, UC-MSC showed lower immunogenicity and a higher capacity for proliferation and differentiation [21, 22]. In the current study, we used UC-MSC as the cell therapy agent and investigated the therapeutic effect of MSC-based treatment in the therapy for pneumonia after HSCT.

In the present study, we found that patients in the MSC group had a better prognosis than those in the non-MSC group. In patients with severe pneumonia, MSC-based treatment promoted significant improvement in the overall cure rate, time to cure, overall survival, and cumulative cure rate. In patients with non-severe pneumonia, MSC-based therapy also significantly improved treatment response in a time-dependent manner, although overall cure rates in the two groups were both 100% and no difference was found. MSCs have been reported to treat various disorders, such as autoimmune, neurodegenerative, and cardiovascular diseases. MSC infusion resulted in improved therapeutic effect and clinical symptoms in patients with graft versus host disease (GVHD), systemic lupus erythematosus (SLE), multiple sclerosis (MS), and Crohn's disease [23–25]. MSC-based treatment is rarely reported in HSCT-related pneumonia, although it was investigated in some studies about other disorders. In a study reported in 2012, MSC was found to be a risk factor for pneumonia-related death after HSCT [26]. In the current study, we focused on children with HSCT for a variety of primary disorders (predominantly thalassemia). In that study [26], primary disorders were mainly leukemia, and patients of all ages (1–71) were included and children were not investigated separately. Besides, not all patients received only one HSCT, and some of them received a second HSCT which increased pneumonia-related death. Furthermore, MSC was used mainly in very sick patients with severe disorders but not randomly in that study, which might cause selection bias. The above differences between the two studies might contribute to the contradictory findings. In other studies, MSCs showed therapeutic potential for acute lung injury (e.g., acute respiratory distress syndrome) and chronic inflammatory disorders (e.g., lung fibrosis and chronic obstructive pulmonary disease) [6, 27, 28]. Our study investigated the efficacy of MSCs for pneumonia following HSCT and first demonstrated that MSC treatment increased the cure rate and reduced the mortality and time to cure of severe pneumonia.

High mortality rates remain the main challenge in the therapy for all severe lung disorders, such as severe pneumonia caused by coronavirus disease 2019 (COVID-19) [29, 30]. Severe pneumonia and acute respiratory distress are the predominate causes of death in patients infected with COVID-19 [29, 30]. Actually, MSC treatment had been reported to improve outcomes in patients with COVID-19 [31, 32]. Along with the above reports, the current study suggested MSC treatment as a promising therapy for severe pneumonia.

MSC might exert its therapeutic effects via several mechanisms. MSCs could home or migrate into damaged tissues via chemotaxis, diapedesis, and adhesion, which ensure that MSCs reach the lung after intravenous injection [33–35]. MSCs have the potential to differentiate into various cell types, which leads to the repair and regeneration of cells and tissues in the target organ [36–38]. MSCs provide significant immunomodulatory effects, which mediate most of the therapeutic effects for severe inflammatory diseases including immune rejection and GVHD in HSCT patients [35, 38]. The immunosuppression and anti-inflammation effects of MSCs also contribute to tissue repair and healing [38–40]. Additionally, Shobha Regmi et al. [41] reported that MSCs adopt pro- and anti-inflammatory roles depending on local microenvironment: MSCs exerted pro-inflammatory effect during early phases of inflammation, whereas its anti-inflammatory effects were more predominant in later phases or worsening condition. It is consistent with the results in our study that the therapeutic effects of MSCs were more significant for severe pneumonia than that for non-severe pneumonia. The detailed mechanisms underlying this behavior need to be explored and clarified in future studies.

Although to our knowledge the current study is the first to investigate the efficacy of MSC treatment in children with pneumonia following HSCT, several limitations should be acknowledged. First, only pneumonia cases were investigated, children with other severe complications post-HSCT was not included. Second, the patients in this study were all recruited from one hospital, and multicenter studies are needed to further confirm our results. In addition, although the sample size in this study was a relatively large one, larger studies with more patients need to be conducted in the future.

## Conclusions

In conclusion, our study revealed that the MSC-based strategy contributes to improvements of therapeutic effect and survival in HSCT children with pneumonia, especially in severe pneumonia cases. Our study suggests MSC treatment as a promising therapy for severe pneumonia. In the future, multicenter studies with larger sample size are encouraged to verify and validate our results.

## Supplementary Information

**Additional file 1: Fig. S1**. Chest CT images of represent cases in MSC group (A) and non-MSC group (B) during the period of treatment for HSCT-pneumonia. (A) CT images of a patient in MSC group whose pneumonia was cured. (B) CT images of a patient in non-MSC group who died from severe pneumonia.

## Data Availability

The datasets used and/or analyzed in this study are available from the corresponding author upon reasonable request.

## Abbreviations

ALD: Adrenoleukodystrophy
AT: Adipose tissue
BM: Bone marrow
CI: Confidence interval
COVID-19: Coronavirus disease 2019
EMR: Electronic medical record
GVHD: Graft versus host disease
GWCMC: Guangzhou Women and Children’s Medical Center
HIES: Hyperimmunoglobulin E syndrome
HSCT: Hematopoietic stem cell transplantation
MPS: Mucopolysaccharidoses
MS: Multiple sclerosis
MSC: Mesenchymal stromal cell
OR: Odds ratio
PKD: Pyruvate kinase deficiency
SLE: Systemic lupus erythematosus
UC: Umbilical cord
