# Supplementary figures and images for: Mesenchymal stromal cell treatment improves outcomes in children with pneumonia post-hematopoietic stem cell transplantation: a retrospective cohort study
Source: Stem Cell Res Ther. 2022 Jun 28;13:277. doi: 10.1186/s13287-022-02960-7 (PMC9241242; doi:10.1186/s13287-022-02960-7)

**A**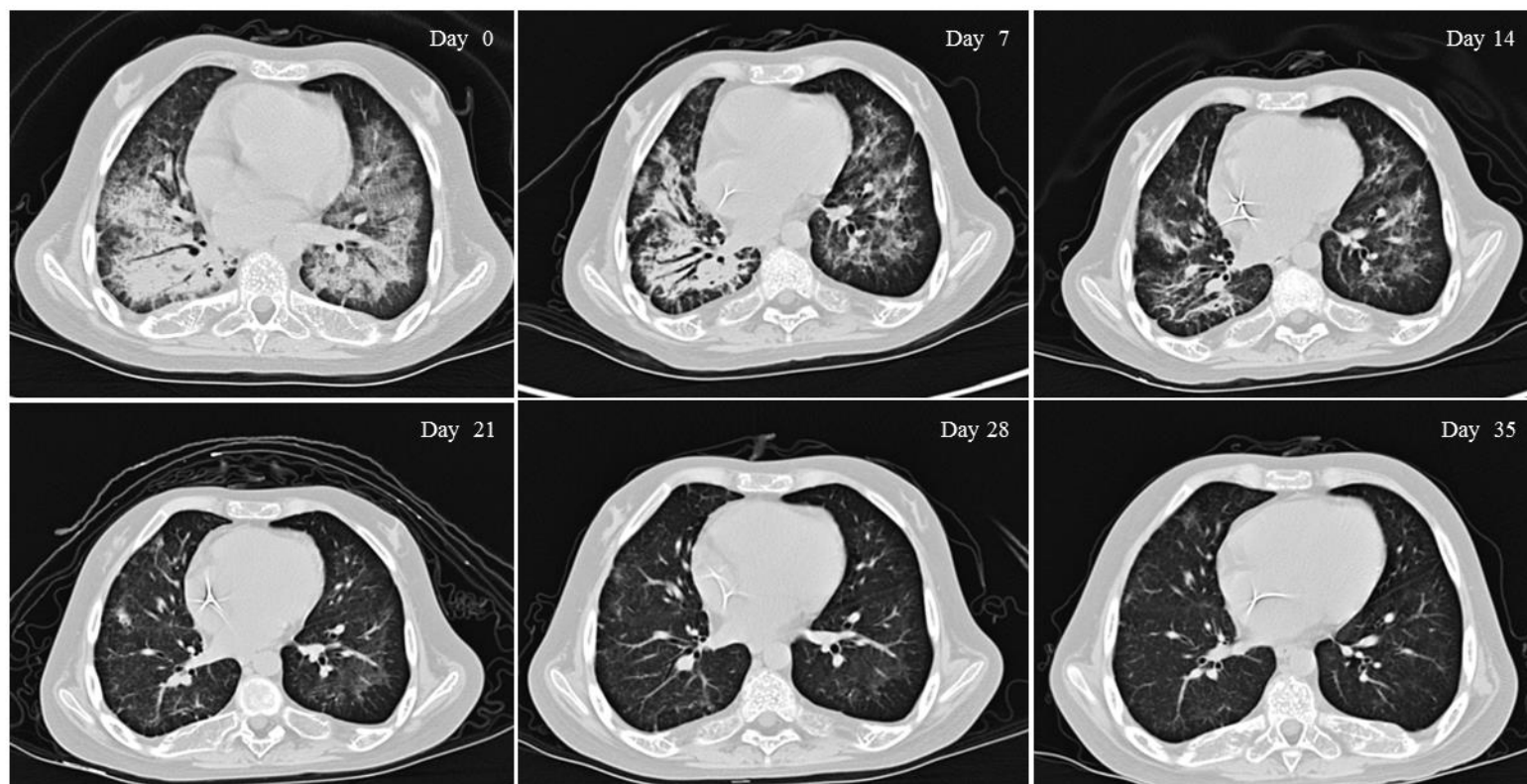**B**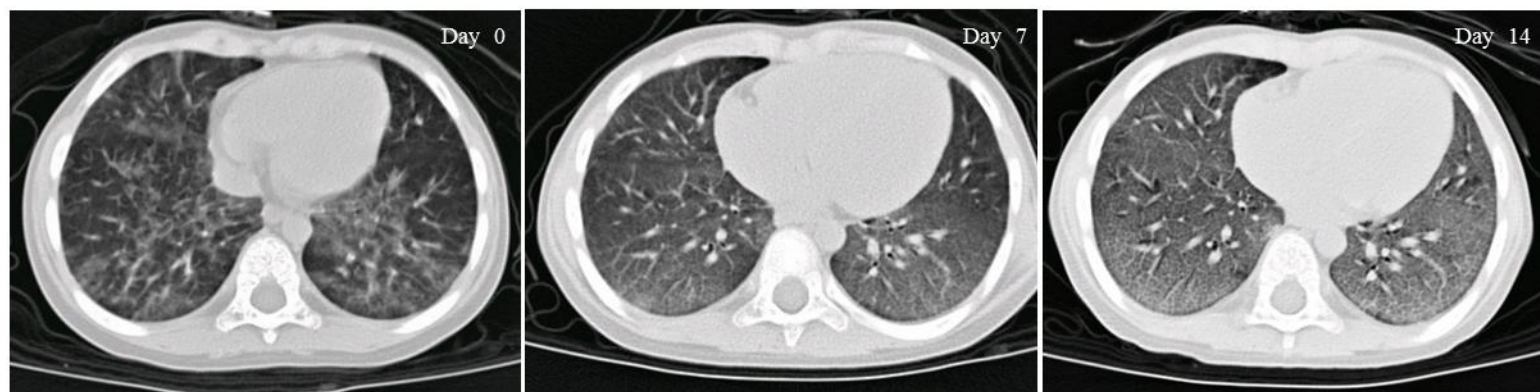

Supplement: Supplementary file 1 — Additional file 1: Fig. S1. Chest CT images of represent cases in MSC group (A) and non-MSC group (B) during the period of treatment for HSCT-pneumonia. (A) CT images of a patient in MSC group whose pneumonia was cured. (B) CT images of a patient in non-MSC group who died from severe pneumonia. [file 13287_2022_2960_MOESM1_ESM.pdf]
